# New Technological Approaches for Dental Caries Treatment: From Liquid Crystalline Systems to Nanocarriers

**DOI:** 10.3390/pharmaceutics15030762

**Published:** 2023-02-24

**Authors:** Marcela Tavares Luiz, Leonardo Delello di Filippo, Jessyca Aparecida Paes Dutra, Juliana Santos Rosa Viegas, Amanda Letícia Polli Silvestre, Caroline Anselmi, Jonatas Lobato Duarte, Giovana Maria Fioramonti Calixto, Marlus Chorilli

**Affiliations:** 1 School of Pharmaceutical Sciences, São Paulo State University (UNESP), Araraquara 14800-903, São Paulo, Brazil; 2 Instituto de Investigação e Inovação em Saúde, Universidade do Porto, 4200-135 Porto, Portugal; 3 School of Dentistry, São Paulo State University (UNESP), Araraquara 14801-903, São Paulo, Brazil

**Keywords:** buccal administration, drug delivery systems, nanotechnology, oral biofilm, remineralization

## Abstract

Dental caries is the most common oral disease, with high prevalence rates in adolescents and low-income and lower-middle-income countries. This disease originates from acid production by bacteria, leading to demineralization of the dental enamel and the formation of cavities. The treatment of caries remains a global challenge and the development of effective drug delivery systems is a potential strategy. In this context, different drug delivery systems have been investigated to remove oral biofilms and remineralize dental enamel. For a successful application of these systems, it is necessary that they remain adhered to the surfaces of the teeth to allow enough time for the removal of biofilms and enamel remineralization, thus, the use of mucoadhesive systems is highly encouraged. Among the systems used for this purpose, liquid crystalline systems, polymer-based nanoparticles, lipid-based nanoparticles, and inorganic nanoparticles have demonstrated great potential for preventing and treating dental caries through their own antimicrobial and remineralization properties or through delivering drugs. Therefore, the present review addresses the main drug delivery systems investigated in the treatment and prevention of dental caries.

## 1. Introduction

It is estimated that oral diseases affect nearly 3.5 billion people. Dental caries is the most common oral disease with prevalence rates being 100% and 80% among 12-year-old adolescents in low-income and lower-middle-income countries, respectively [1]. According to the World Health Organization (WHO), untreated dental caries (tooth decay) in permanent teeth is the most common health condition [2]. It is a multifactorial chronic infection of the oral cavity caused by bacterial plaque [3], an age-related and a life-long disease [4], and is associated with a substantial health and economic burden [5].

Dental caries is caused by the production of acid originating from bacteria fermenting carbohydrates leading to demineralization of the dental enamel and the formation of cavities [6]. The main clinical symptoms and signs of dental caries are spontaneous pain, nighttime pain, referred pain, thermal sensitivity, and percussion pain [7].

The burden of dental caries remains a global public health challenge and requires new tools for its treatment. The development of a clinically appropriate, safe, and effective delivery system for buccal administration represents a pivotal point for the treatment of caries [8].

**Figure 1:**
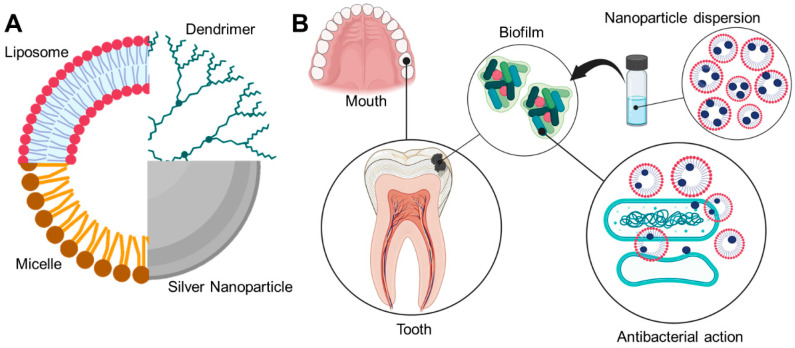
Illustration of the application of (**A**) different drug delivery systems on (**B**) caries prevention and treatment. Figure created with BioRender.com.

The combination of drug delivery and controlled release systems (e.g., polymeric nanoparticles, metallic nanoparticles, and liposomes) with the research and development around caries prevention has gradually emerged due to several benefits (Table 1) [9]. These systems can entrap substances/drugs and use the advantages of small size and better penetration [10]. Facilitating prolonged residence time in the buccal cavity and reducing the emergence of drug-resistant phenotypes [11]. An example of a promising drug delivery system for buccal administration is the liquid crystalline system (LCS) due to the possible changes in the viscosity of formulations promoted by the mesophase transition triggered by the incorporation of water from the saliva, which can increase the residence time of the formulation in the mouth and provides drug-controlled release [12]. In addition, they can promote the controlled release of drugs and protect active ingredients from thermal degradation and photodegradation, improving formulation stability [13]. Moreover, the use of polymers in LCS and o other drug delivery systems can favor characteristics such as shear thinning, thixotropy, and bioadhesive making them excellent formulations for dental delivery and treatment of caries (Figure 1) [8,12,14,15]. In this context, the potential of nanosystems have been investigated in some clinical trials (Table 2).

This review summarizes and discusses the liquid crystal systems and the various novel nanotechnology-based drug delivery systems used to prevent and treat dental caries. We will start with a brief discussion of the pathophysiology of the disease with emphasis on the formation of biofilms and proceed to further discussion on the delivery systems.

## 2. Physiopathology and the Formation of Biofilms

The definition of biofilm is the oriented aggregation of microorganisms joined by an extracellular polymeric matrix [38,39], with only 5–25% composed of bacterial cells and the remainder filled with glycocalyx produced by themselves [40]. The bacterial organization in biofilms is the most common way found in nature, it is estimated that about 95% of existing bacteria are in biofilms. This way of organizing allows microorganisms to adhere and multiply on surfaces and the formation of different chemical components and nutrient gradients that allow the creation of microenvironments favorable to the proliferation of each type of microorganism [41].

The oral cavity is colonized by more than 700 species of bacteria [42,43] that are present in teeth, gums, periodontal space, and throughout oral mucosa. Each species selects the site according to the characteristics that the site can provide, such as access to oxygen and nutrients [44]. Most of these bacteria live in commensalism with the host, which is essential for health maintenance [39]. The way in which the biofilm is organized and the competition between the different species is crucial for the balance of the health condition or oral tissue disease [39,41]. Therefore, it is necessary to know how microorganisms adhere, organize, and multiply on the dental surface for developing strategies for the prevention and treatment of caries.

**Figure 2:**
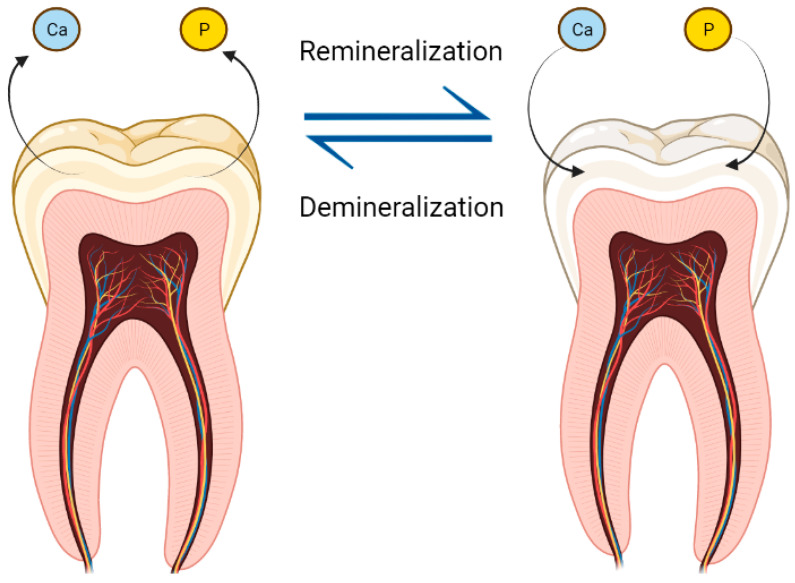
Illustration of the demineralization and remineralization processes. Figure created with BioRender.com.

To initiate the adhesion of the biofilm to the surface of the dental enamel, there is first the adsorption of a thin organic, protein and acellular layer formed by glycoproteins from saliva, which is known as acquired pellicle. This pellicle is responsible for the lubrication and hydration of dental tissues, in addition to participating in the de/remineralization processes (Figure 2) and playing a fundamental role in the adhesion of bacteria to the tooth surface [45]. This first stage of acquired pellicle formation begins instantly when saliva touches the enamel surface [46,47]. The first layer is formed due to the strong tendency for calcium ions to crystallize, leaving the remaining phosphate ions free to deposit on the enamel surface (Hannig, 1999). As result of this deposition, the enamel surface becomes negatively charged, which will be covered with a positive layer of calcium ions. After that, a fraction of the proteins present in the saliva will be adsorbed to this double layer ions by electrostatic interactions [48]. The adhesion of bacteria, pathogenic or not, to surfaces is directly linked to the adhesion of bacterial exoenzymes to proteins present in this layer, such as agglutinin, amylase, fibrinogen, fibronectin, or mucins [49].

In the first stages of biofilm formation, during the first 6 h, are found some microorganisms, such as *Streptococcus*, *Neisseria*, *Rothia*, *Actinomyces*, or *Veillonella*. Subsequently, other microorganisms initiate *secondary* colonization (e.g., *Fusobacterium nucleatum*, *Treponema* sp., *Porphyromonas gingivalis*, or *Aggregatibacter*) [50]. In the same period, the beginning of the development of the extracellular matrix also occurs, which plays a key role in the formation, pathophysiology, and virulence of biofilms [49,51,52].

The extracellular matrix of biofilms plays an essential role as support structure for the formation of the three-dimensional biofilm, in addition to regulating metabolic exchanges, signaling between microorganisms, and enabling the supply of nutrients [53,54]. This matrix is formed by polysaccharides produced by exoenzymes originating mainly from *Streptococcus mutans* adhered in the first stages of colonization of cariogenic biofilms [55]. Although *S. mutans* is not the most present microorganism in biofilms, it is important to the biofilm organization process, due to the production of exoenzymes, such as glucosyltransferase. This enzymes adsorbed on the surfaces synthesize exopolysaccharides (EPS) from sucrose and starch [52,55]. These EPS participate in the process of adhesion of the biofilm to the acquired film, as well as in the structuring of an extracellular matrix with low solubility, promoting a favorable surface for the accumulation and formation of a more complex biofilm [56].

This complexity can define the biofilms pathogenicity, the production of acid by bacterial metabolism may not be sufficient to disease development, but the formation of acidic microenvironments with sites protected from the buffering action of saliva can cause disease [57]. Therefore, along the biofilm, it is possible to notice a pH gradient, with acidic sites colonized by grouped acidogenic bacteria [56]. These bacteria use both the external carbohydrates influx and the matrix polysaccharides themselves as a source of carbohydrates for the development of metabolic activities [55,58].

All exchanges and interactions make the biofilm not static, it is a structure in constant remodeling [55,57]. During the initial formation, there is a large production of the matrix, but it is also reabsorbed in certain places, this process changes the topography and biomechanical properties of the matrix and corroborates the dispersion of microorganisms [59]. An example of this process is the remodeling that occurs in cariogenic biofilms due to the presence of glucanohydrolases and fructanases, enzymes capable of degrading some polysaccharides in the extracellular matrix. These enzymes are produced by the microorganisms themselves, as well as the enzymes responsible for glucan synthesis, and these two processes may be occurring at the same time [60].

The formation of caries lesions is directly linked to the production of acids that cause demineralization of the dental surface [61]. Therefore, in addition to the importance of the bacterial composition and its organization in the extracellular matrix for the pathogenicity of the oral biofilm, the interaction between the host’s diet and the microorganisms present in the biofilm is also fundamental for the development of the disease. The role of *S. mutans* is given by its ability to use carbohydrates present in the host’s diet and create an insoluble polymeric extracellular matrix that protects acidogenic sites so that other species of bacteria present can produce more acid, keeping away from the buffering capacity of saliva [51]. Thus, we note that the abundance of fermentable carbohydrates in the host’s diet is what makes the process of biofilm formation complex and more pathogenic [52].

Finally, the strategies used for the prevention and treatment of caries and biofilm formation must take into account the way the biofilm adheres to the dental surface, how it is structured, the bacterial composition, as well as the complexity of the interaction of microorganisms with each other and with the components of the extracellular matrix.

## 3. Drug Delivery Systems Used in the Prevention and Treatment of Caries

### 3.1. Organic Systems

#### 3.1.1. Liquid Crystalline Systems

The first relates of liquid crystalline systems (LCS) date from 1888 by the Austrian botanist Friedrich Reinitzer. LCS are systems possible thanks to an “intermediate” state of the matter; these drug delivery systems have the fluidity of a liquid but the crystalline internal structure of a solid. This “intermediate” state can also be called mesophase. Despite many other uses, LCS are particularly interesting for drug delivery in dentistry (Table 3) because these systems can enhance the solubility of poorly water-soluble drugs, besides promoting a controlled release of molecules and adhesive properties, which is a key factor that contributes to an improved therapeutic effect. In addition, LCS are biocompatible, cheap, and easy to produce (i.e., direct mixing of ingredients), and have good physical-chemical stability. LCS are composed of rigid molecules with polar groups that exhibit relatively strong dipole–dipole or dipole–induced dipole interactions, such as lipids, polymers, and surfactants. Depending on the proportion and/or type of material used in their fabrication, they can form distinct mesophases, that can influence the release rate and solubility of drugs, as well as other properties [62,63].

In this context, Aida and coworkers developed a liquid crystalline precursor to deliver the cationic antimicrobial peptide β-defensin-3 peptide fragment (D1–23) for anti-adhesion purposes against *S. mutans* and its proliferation in dental biofilms [15]. The system was prepared using oleic acid, polyoxypropylene-(5)-polyoxyethylene-(20)-cetyl alcohol, Carbopol^®^ 974P, and Carbopol^®^ 971P. Experiments using artificial saliva showed different mesophase formation with different proportions of saliva. The addition of artificial saliva also promoted an increase in viscosity and bioadhesiveness of the system, desirable to achieve high retention time in the oral cavity for prolonged antimicrobial effect. The formulation exhibited a time-dependent antimicrobial effect, indicating that the incorporation of D1-23 into the LCS did not impair its antimicrobial properties, but required a longer time to act, compared to the D1-23 solution. In vitro cytotoxicity assays using the HaCaT cell line indicated that D1-23 solutions are very cytotoxic, but not when incorporated into the LCS at the same concentrations, suggesting another important feature for a safer and localized treatment of caries.

Calixto and coworkers also explored the potential of LCS to deliver an anti-microbial peptide (p1025) to treat dental caries. This peptide is capable of inhibiting the binding of *S. mutans* onto the teeth’ surface. The LCs were obtained using tea tree oil as the oil phase, polyoxypropylene-(5)-polyoxyethylene-(20)-cetyl alcohol as the surfactant, and a 0.5% aqueous dispersion of polycarbophil polymer, as a strategy to improve bioadhesion. The polarized light microscopy and small-angle X-ray scattering evidenced the formation of hexagonal phases and microemulsions that exhibited anticaries and bioadhesive properties and provided a protective environment to p1025 at the site of action, in addition to prolonging its contact with the teeth. Together, the data shown in this study reinforce the potential of LCS as a novel platform for dental caries treatment [8].

In another study, Calixto and coworkers [64] developed a liquid crystalline precursor system of oral in situ gelling to deliver p1025, using polyoxypropylene-(5)-polyoxyethylene-(20)-cetyl alcohol, oleic acid, and Carbopol C974P. The formulations obtained presented strongly bioadhesive hexagonal mesophases. The authors investigated the formulations’ anti-biofilm properties using *S. mutans*, and results showed that p1025 alone had no anti-biofilm activity, while LCS showed equivalent biofilm eradication compared to chlorhexidine (standard treatment). The LCS formulations have also proven to have low cytotoxicity, promoting in vitro HaCaT cell viability up to 70%. These results encourage further investigations of bioadhesive LCS containing p1025 as a preventive tool for biofilm eradication.

Bernegossi and coworkers also explored the potential of LCS to deliver peptides of medical importance. They developed a LCS containing the decapeptide KSL-W, which has interesting antimicrobial and antibiofilm properties. LCS were obtained using oleic acid, polyoxypropylene (5) polyoxyethylene (20) cetyl alcohol (PPG-5-CETETH-20), and a 1% poloxamer 407 dispersion, which originated lamellar mesophases; formulations with increased water content showed superior bioadhesiveness. In vitro studies using human saliva samples were grown to form a multispecies biofilm model, and KSL-W-loaded LCS completely eradicated colony-forming units (CFU), as well as the negative control, chlorhexidine. The free peptide-treated plates presented bacteria growth (9% CFU). These results reinforce the potential of LCS as alternative platforms to deliver antimicrobial agents and treat oral biofilms, including the multispecies [14].

LCS are also useful to deliver conventionally used antibiotics, such as poly(hexamethylene biguanide) hydrochloride (PHMB), a cationic amphiphilic molecule with a broad antimicrobial spectrum against both Gram-positive and -negative bacteria, as demonstrated by Souza and coworkers [86]. LCS were made using a monoacylglycerol (Myverol 18–99^®^) and water, producing LCS of lamellar and cubic mesophases. The systems also presented a sustained in vitro release of PHMB for up to 24 h. The in vitro antimicrobial activity was assessed using *S. mutans* and *C. albicans*, and the data confirm that LCS containing PHMB have superior antimicrobial effects compared to PHMB in solution, in a dose-dependent way. The addition of PHMB in LCS increased the bioadhesive force of LCS, suggesting that lamellar and cubic LCS are promising vehicles for the buccal administration of antibiotics, promoting prolonged action and efficient local delivery.

Due to increased retention time onto the teeth surface and sustained release properties, LCs seems to be particularly useful in photodynamic inactivation (PDI) of oral biofilms. With that in mind, Reina and coworkers developed curcumin-loaded liquid crystalline precursor system for PDI of biofilms. LCS were obtained using Carbopol 974P, oleic acid and PPG-5-CETETH-20. In vitro release studies confirm a controlled release behavior of LCS. Despite presenting different PDI efficacy among biofilm samples (related to different volunteers’ microbiota, salivary flow, salivary pH, and diet), the LCS containing curcumin presented the highest CFU counting decrease, evidencing that LCS associated with a photosensitizer can be useful to eradicate oral biofilms, promoting protection for the photosensitizing agent as well as a sustained and local effect [65].

#### 3.1.2. Liposomes

Liposomes are lipid vesicles widely studied in the nanomedicine field, including for dentistry applications (Table 3) [87]. They were first described in the 1970s, and since then, their attractive features have gained special attention for improving the treatment of several diseases. The liposomes are formed by an inner aqueous core surrounded by at least one lipid bilayer, in which the lipid aliphatic chains interact among themselves, and the lipid polar heads interact with the aqueous phase. They are composed of synthetic or natural phospholipids that have two tails. However, other components can be added to their formulation to improve some characteristics required in the research, such as permeation and mucoadhesive properties [88,89,90]. The main advantages of these systems are their low toxicity, high capacity to encapsulate both lipophilic and hydrophilic drugs, and their possibility to be modified with other components and generate nanosystems with different physicochemical and biological features [88,91].

Thus, the advantages of these delivery systems have made them attractive for the treatment of dentistry diseases. Their use have been described for prevention of caries and periodontitis through the disruption of oral biofilms [66,67,92,93,94], reduction in acid erosion [95], and enhancement of the biomineralization for dental tissue regeneration [68,96].

The control of oral biofilms requires a prolonged residence time of nanosystems in the oral cavity. To provide this feature, cationic liposomes and liposomes covered with polysaccharides to form bioadhesive systems are two interesting strategies investigated [97]. Harper and coworkers (2018) studied the influence of positive and neutral α-tocopherol liposomes in the control of bacterial saliva biofilm growth. The results of positive liposomes showed a greater increase in adsorption to hydroxyapatite, the main constituent present in the teeth, when compared with neutral liposomes, demonstrating the greater potential of cationic vesicles to adhere to the teeth. Furthermore, positive vesicles were able to enhance the growth inhibition of oral bacteria due to its high bioadhesivity and penetration properties, with a significant smaller biofilm height (38 ± 7 µm) when compared with vehicle control (about 75 µm) [93].

Another study conducted by Yamakami and coworkers also evaluated the influence of the liposome’s charge (cationic, anionic, and neutral) to increase their oral retention time. The author encapsulated a bacteriocin, called nisin, into the liposomes and investigated their antimicrobial activity against *S. mutans*. The results indicated that nisin-loaded cationic liposomes exhibited sustained release, achieving 41% of releasing upon 2 h of study, whereas at the same time the control liposomes released 80% of nisin. Furthermore, in vitro assay indicated a higher antimicrobial activity of cationic liposomes when compared with the control group (2-fold higher), which was greater than non-ionic and anionic liposomes (1.3- and 0.6-fold, respectively). The results demonstrated that the electrostatic interaction between liposomes and bacterial membranes can be a great strategy to enhance oral retention and improve the activity against microorganisms [66].

Zhou and coworkers (2018) evaluated the treatment of oral biofilms employing pH-responsive doxycycline-loaded liposomes. The author produced liposomes coated with quaternary ammonium chitosan (TMC) to increase the oral retention time and their adhesion with the biofilm. Moreover, TMC was used due to its antimicrobial activity and ability to destabilize liposomes in acid pH biofilm environments, commonly found on biofilms. This destabilization at acid pH can contribute with a higher release rate of doxycycline in the target site. The in vitro release study at pH 4.5 and 6.8 proved this pH-triggered doxycycline release, with t_1/2_ = 0.75 h and 2.3 h, respectively. Confocal imaging indicated the potential of this formulation to adhere to the surface of hydroxyapatite. Furthermore, the antimicrobial activity study indicated that the developed system showed a significant inhibition rate against *P. gengivalis*, with a lower biofilm mass, demonstrating its great potential to treat oral plaque biofilm [67].

The prevention of acid erosion of hydroxyapatite matrix using alkali-loaded thermosensitive liposomes (Tris-Lipo) was analyzed by Chong and coworkers (2020). The author developed a nanosystem able to sustain the release of alkali buffer (Tris buffer) at 36.5 °C to neutralize the acid environment that is responsible for hydroxyapatite erosion and demineralization. The release rate of Tris-Lipo showed a prolonged profile with the ability to increase pH above the dentine erosion critical pH (value of 5.5) after 1.5 h of incubation, which was effective against the acid erosion by lactobacilli in the oral cavity. During in vitro experiments, the developed formulation was able to reduce the average eroded volume of hydroxyapatite when compared with the untreated control, with an eroded volume of 6.1%. The demineralization volume was also reduced significantly when Tris-Lipo was evaluated. Thus, this system showed a potential to inhibit dentine erosion and demineralization caused by dentine acidification by lactobacilli [95].

In response to demineralization generated by trauma or caries, liposomes can act as a delivery system to carry substances that promote dental restoration. Melling and coworkers (2018) developed liposomes to load demineralized dentin matrix (DDM) extracted from noncarious dentine, which has the potential to promote dentin regeneration. The results indicated that DDM and DDM-loaded liposomes increased osteocalcin expression about 12- and 26-fold, respectively, indicating the potential of DDM-loaded liposomes to regenerate dentinogenesis. Furthermore, the mineralization assay suggested that control liposomes (without DDM) were able to increase the mineralization process. This result can be related to the presence of phosphatidylserine in liposome composition that can bind to calcium and induce mineralization. DDM-loaded liposomes showed to be more effective than control liposomes, which can be related to DDM and phosphatidylserine synergism, demonstrating the potential of this formulation in dental restorative processes [68]. This enhancement of mineralization by phosphatidylserine was also demonstrated by Park and coworkers (2017) when the author analyzed the potential of liposomes composed by phosphatidylserine to stimulate dentin formation in human dental pulp cells [96].

#### 3.1.3. Nanoemulsion

Nanoemulsions are a heterogeneous dispersion composed of two immiscible liquids, surfactant, and co-surfactant, in which nanodroplets of one of the liquids (internal phase) are dispersed throughout the other liquid (external phase). The nanodroplet sizes range from 20 to 200 nm and can be stabilized by different surfactants. The mixture of oil, water, surfactant, and co-surfactant gives thermodynamic stability to this nanosystem, reducing the occurrence of flocculation, coalescence, sedimentation, and creaming. In addition, nanoemulsions are greater absorbed in the tissue owing to their high surface area [21,22]. These features make this nanosystem gain more attention in dentistry, including for oral biofilm remotion [11].

Ramalingam and coworkers investigated the antimicrobial activity of nanoemulsion on planktonic *S. mutans*, *Lactobacillus casei*, *Actinomyces viscosus*, and *C. albicans*. The nanoemulsion was composed of soybean oil, Triton X-100, and cetylpirydinium chloride (CPC). The CPC is a quaternary ammonium salt approved by the Food and Drug Administration (FDA) as an effective antiplaque agent. This formulation was able to inhibit microorganisms’ concentration 9- to 27-fold greater than chlorhexidine solution, with a higher MIC observed for *S. mutans* and *A. viscosus*. The nanoemulsion has also shown higher bactericidal activity than chlorhexidine solution in mixed culture strains. In addition, the adherence assay indicated that the developed nanoemulsion inhibited (94.2–99.5%) the adherence of cells to the glass surface and reduced bacterial counts in mixed cultures biofilms. Thus, the anti-cariogenic activity of the developed formulation indicated that nanoemulsion containing CPC is a potential medication to prevent caries [69].

Li and coworkers developed a nanoemulsion for improving the antimicrobial activity of chlorhexidine acetate against *S. mutans* in vitro and in vivo. The chlorhexidine acetate-loaded nanoemulsion was composed of isopropyl myristate, Tween 80, and propylene glycol. The nanosystems had an average size of 63.13 nm and the ability to reduce the MIC and MBC two times more than chlorhexidine solution. In vitro studies have also shown a fast-acting bactericidal activity of chlorhexidine acetate-loaded nanoemulsion, promoting 95.07% death within 5 min, while chlorhexidine caused 73.33%. The in vivo studies using Sprague Dawley rats indicated the superior capacity of the developed nanoemulsion to reduce oral *S. mutans* growth and inhibit biofilm formation, compared with chlorhexidine. The results demonstrated that chlorhexidine acetate-loaded nanoemulsion has the potential to be applied in clinical for preventing and treating dental caries [70].

Jeong and coworkers produced cinnamon essential oil nanoemulsion to evaluate this antibacterial and antibiofilm effect against oral biofilms. The nanoemulsion was composed of cocamidopropyl betaine, as a surfactant, and cinnamon essential oil, showing an average size of 207.2 nm. The in vitro results demonstrated that cinnamon essential oil nanoemulsion had a similar effect to chlorhexidine in terms of inhibiting the maturation of early and mature biofilms. Furthermore, the nanosystems suppressed the growth of caries-causing aciduric bacteria in the same way that chlorhexidine, a gold-standard antimicrobial agent, demonstrates the potential of this nanoemulsion against oral biofilms [71].

According to the studies found in the literature, nanoemulsion shows great potential to be applied in clinical for preventing and treating dental caries, which can be related to their ability to deliver hydrophobic antimicrobial agents and permeate oral biofilms due to their high surface area. However, until the present moment, few studies have been published using this nanosystem for preventing and treating dental caries. Thus, clinical studies are needed to better understand the potential of nanoemulsions in the dentistry field.

#### 3.1.4. Polymeric Nanoparticles

Polymeric nanoparticles (PNs) are colloidal systems consisting of polymers that can be synthetic or natural and they can have their structure denominated by nanocapsules (polymeric walls with a core made up of oil or water) and nanospheres (they are made up of polymeric matrices and have no core) as well as medium size up to 1000 nm [98,99]. There are several methods for obtaining PNs, including polyelectrolytic complexation, ionotropic gelation, emulsification solvent diffusion, solvent evaporation, and nanoprecipitation [100,101,102,103]. PNs have a vast advantage, such as increasing drug stability (protection against oxygen, moisture, and light), improving drug solubility, and controlled release. In addition, some polymers have mucoadhesive properties [24].

Ikono et al. (2019) developed chitosan (CS) and tripolyphosphate (TPP) nanoparticles using the ionotropic gelation method for the treatment of caries caused by *S. mutans*. Transmission electron microscopy analyzes showed that the PNs had an average size of 20–30 nm and an almost spherical morphology. In vitro, biological tests showed a decrease in cell viability mainly at the highest concentration of NPs (45%). The CFU/mL results for the single *S. mutans* species after 3 h incubation yielded a CFU/mL of 5 log10 compared to a control which was nearly 8 log10. Therefore, this study showed a promise antimicrobial activity of PNs for biofilms in the oral cavity because the positive charge of CS promotes a strong interaction with the teichoic acids present in the cell wall of *S. mutans*, breaking the cell membrane and, due to the reduced size of the NP, they can penetrate more efficiently in biofilms. However, more tests should be performed to improve the study [72].

Aliasghari and coworkers also developed chitosan PNs for the treatment of caries caused by *S. mutans*, *S. salivarius*, *S. sobrinus*, and *S. sanguis*. The PNs were tested in concentrations of 5, 1.25, and 2.5 mg/mL obtaining an average size of around 10 to 16 nm depending on the concentration. The results of MIC (minimum inhibitory concentration) assays of NPs for *S. mutans* and *S. sanguis* were 1.25 mg/mL and for *S. salivarius* and *S. sobrinus* were 0.625 mg/mL. The MIC values of the CS-PNs for *S. mutans* and *S. sanguis* were 1.25 mg/mL and for *S. salivarius* and *S. sobrinus* were 2.5 mg/mL. On the other hand, the MBC (minimum bactericidal concentration) values were 0.625 mg/mL for *S. mutans*, *S. salivarius*, and *S. sobrinus*, and a value of 0.312 mg/mL was obtained for *S. sanguis*. All samples tested with a concentration of 5 mg/mL decreased the biofilm formation of *S. mutans* by 88.4%, *S. salivarius* by 93.4%, *S. sobrinus* by 78.9%, and *S. sanguis* by 72.6%. Therefore, this study showed potential results for the use of chitosan NPs through the interaction of fillers with the bacterium, suggesting a promising treatment for dental diseases [73].

Maghsoudi and coworkers developed nanoparticles of CS, alginate (AL), and starch (ST) incorporated with curcumin through the desolvation method for the treatment of caries caused by *S. mutans*. The results of in vitro release showed that curcumin is released after 12 h in quantities of 63.5% (PNsCS), 36.34% (PNsAL), and 11.1% (PNsST), after 24 h it reached 75.3%, 51.2%, and 19.3%, respectively, and after 96 h they were 92.8%, 51.4%, and 73.4%, respectively. The results of MIC assay for CS PNs against *S. mutans* were 0.114 mg/mL and for AL and ST PNs were 0.204 mg/mL, whereas, compared to MIC for free curcumin (0.438 mg/mL), PNs show better properties inhibitory. In the inhibitory effect assays on biofilm at pH 5 and 7, it was noted that curcumin loaded in PNs had a greater inhibitory effect compared to pure curcumin at both pHs. Pure curcumin obtained a 67.38% inhibition value compared to the tested NPs, being 95.49% (PNsCS), 95.28% (PNsAL), and 89.48% (PNsST) at pH 5, unlike pH 7 where values of 93.78% were obtained (PNsCS) and 93.13% (PNsAL) and 99.38% (PNsST) compared to pure curcumin (59.97%). The authors suggest that the reduced size of the nanoparticles leads to a greater diffusion of the drug and that curcumin inhibits the activity of the enzyme sortase A whose main function is to maintain the structure of the wall of *S.mutans*. For this reason, this study shows that these NPs are incredible potential for the treatment of dental caries [104].

The work of Cavalcante and coworkers was to develop chitosan nanoparticles (CSNPs) incorporated with chloroaluminum phthalocyanine (ClAlPc) through the inotropic gelation preparation method to evaluate photoinactivation mediated through antimicrobial photodynamic therapy (aPDT) against biofilms of *S. mutans*. The characterization revealed that the nanoparticles were found at the nanometer scale (size values around 300 nm with polydispersity index less than 0.3 and zeta potential greater than +30 mV) and ClAlPc was also adequate through photophysical and photochemical parameters. Treatment with aPDT mediated by ClAlPc + CSNPs showed a significant reduction in S. mutans viability (1 log10 CFU/mL reduction) compared to the negative control (PBS, *p* < 0.05). Scanning electron microscopy assays revealed a change in biofilm morphology after treatment of bacteria with aPDT ClAlPc + CSNPs and a reduction in the biofilm formed by short cell chains was verified, suggesting a reduction in the number of adherent bacteria and lower production of extracellular matrix. Therefore, this study showed an interesting strategy for the treatment of caries [74].

#### 3.1.5. Hydrogels

Hydrogels (HG) are polymeric networks cross-linked by physical-chemical bonds, forming a mesh in the three-dimensional structure with a size between 1 and 1000 nm [105]. These polymeric networks can consist of natural polymers (chitosan, alginate, cellulose, starch, guar gum, collagens, proteins, acacia, and acid) or synthetic polymers (polyacrylic acid, polyacrylamide, polyvinyl pyrrolidone, acrylic acid, methacrylic acid, N-isopropyl acrylamide, N-vinyl-2-pyrrolidone, etc.) soluble in water [106]. Due to their hydrophilic structure, they are extremely biocompatible in biological fluids and non-toxic. In addition, they can present mucoadhesion depending on the polymer used and modulated for a controlled drug release [25]. HGs can be obtained by physical methods, such as stereo complex formation, H-bonding, ionic interaction, and freeze–thawing. Furthermore, chemical methods are also used, such as radical polymerization, condensation reaction, and high energy radiation [107].

Ren and coworkers developed chitosan hydrogels (CS-HG) incorporated with QP5 peptide (CSHG-QP5) for the treatment of dental caries. For this, the activity of CS-HG and CSHG-QP5 on *S. mutans* biofilm was assessed. The MIC/MBC value of HGCS-QP5 towards *S. mutans* was 5 mg/mL. On the other hand, the free QP5 does not show activity. The adhesion tests demonstrated that HGCS-QP5 inhibited the adhesion of *S. mutans* in 95.43% compared to the free QP5, which obtained an inhibition of 27.97%. For the experiment with the biofilm, HGCS-QP5 still presented better results when compared with free QP5, indicating almost 100% inhibition of the biofilm formation. Moreover, in the remineralization assay, the developed formulation showed 50% surface micro-hardness recovery, less mineral loss, and more mineral content. Thus, it is noted that the incorporation of active substances in systems such as hydrogel improves antimicrobial activity and is a promising treatment against dental caries [75].

Ashrafi and coworkers developed chitosan nanogel incorporated with essential oils of *Mentha piperita* (MPEO) for the treatment of *S. mutans* biofilm. The nanogel obtained a size of 575.6 nm, polydispersity index of 0.584, and zeta potential around +35 mV. The scanning electron microscopy and energy dispersive X-ray confirmed the encapsulation of MPEO in the nanogel. The biofilm susceptibility assessment showed that the nanogel-MPEO and the free nanogel inhibited 57% and 35% of *S. mutans* cells, respectively. The gene expression assay showed a negative regulatory effect on eight genes involved in biofilm formation and virulence factors. Thus, this study demonstrated that MPEO in nanogel is a promising tool in the dental field [76].

Tomczyk and coworkers developed sodium carboxymethylcellulose HG containing dry tormentil extract for antimicrobial evaluation in *Streptococcus mutans* biofilm. This study also evaluated the mucoadhesion, viscosity, and texture properties of this formulation, which showed that HG in the highest concentration of tormentil extract (10 mg/mL) obtained greater mucoadhesiveness and viscosity, besides adequate values of hardness, cohesiveness, and consistency. The microbiological results against the *S. mutans* biofilm indicated a significant reduction in biofilm formation, this reduction occurred in a dose-dependent manner until total inhibition at the final concentration of 2 mg/mL of the extract. Therefore, this study showed promise as an antimicrobial activity for *S. mutans* [77].

Silva Junior and coworkers (2016) developed papain-based hydrogels incorporated with methylene blue, a photosensitizer (PapaMBlue), for the treatment of dental caries associated with antimicrobial photodynamic therapy (aPTD). The parameters for aPDT are an LED light source with a power of 149 mW/cm^2^, a wavelength of 660 nm, and an energy dose of 150 and 200 Jcm^−2^. The results show that PapaMBlue produced significantly more reactive oxygen species (ROS) than free methylene blue. The antimicrobial activity of the developed formulations was assessed using S. mutans biofilm. The values showed that in the presence of light within a light dose of 200 J/cm^2^, the free MB at 20 μM, PapaMBlue at 10 μM, and PapaMBlue at 20 μM groups had a reduction in 2-logs of CFU. Therefore, this study showed significant antimicrobial activity of MB associated with HG when exposed to irradiation, demonstrating this potential in aPDT [78].

#### 3.1.6. Dendrimers

Dendrimers are synthetic polymeric nanostructures that were discovered in the 1970s through to the 1990s. Their name is derived from the Greek word dendron, which means “tree” or “branch”, referring to the branched structure of dendrimers, that have a hydrophobic interior and hydrophilic surface. The dendrimers’ structure can be didactically divided into three main parts: a central core, building blocks (forming the branches), and varied functional groups composing the surface. This structure is obtained by a drug encapsulated in the core, with repeating concentric layers of polymer arranged geometrically. Some advantages of dendrimers are the biodegradability, biocompatibility, sustained drug release, and multi-functional properties achieved by easy surface modifications, being able to increase tissue targeting and retention time. Dendrimers also behave as biomimetic globular structures due to their small size (<10 nm) and uniform shape and size. The most extensively used polymers in dendrimers in the dentistry field are polypropylene imine (PPI) and polyamidoamine (PAMAM). In dentistry, the dendrimers represent a promising tool for enhancing of mechanical properties of adhesives and resin structures as drug delivery platforms for antimicrobial drugs for the treatment of periodontal diseases and in peripheral dental implant areas and in dental biomaterials for biomimetic remineralization of enamel and dentin (Table 3) [85].

The infection on dentine hard tissues such as caries demands a clinical approach where the structural integrity and disinfection requirements must be considered. With that in mind, Zhou and coworkers [54] developed a dendrimer based on a disinfectant (triclosan), using a carboxyl-terminated PAMAM polymer for simultaneous treatment of caries and remineralization of human dentine, inducing hydroxyapatite (HA) production with similar characteristics to the natural dentine. The carboxyl group promoted adhesion to the dentine surface strong enough to resist rinsing with deionized water, indicating appropriate adhesion for local treatment. MTT assays using the L929 (mouse fibroblast) cell line demonstrated low cytotoxicity, highlighting the biomedical potential of the dendrimer. In vitro remineralization of dendrimer-coated dentine was investigated in artificial saliva. After 4 weeks, the results suggest that triclosan-loaded dendrimers promoted almost full remineralization of surface and dentine tubules, in a concentration-dependent way, while conventional dendrimers (without triclosan) did not. The in vitro release profile of triclosan was sustained for up to 48 h. These results suggest the great potential of dendrimers for delivering triclosan to stimuli dentine remineralization.

Also exploring PAMAM polymers, Tao and coworkers developed honokiol-loaded dendrimers (PAMH) as an approach to simultaneously treat bacterial infections and enamel demineralization [55]. Honokiol is a plant-derived insoluble antibacterial agent, which can inhibit the growth of several cariogenic pathogens and reduce the acid production by cariogenic bacteria. However, its low water solubility limits its use in the clinic. In cariogenic pH (acidic, ~5.5), PAMH showed a sustained release, desirable to achieve a long-term local treatment. PAMH demonstrated biocompatibility with human oral keratinocytes, after 24 h exposure, indicating its potential to be applied in clinical treatment. The PAMH formulation also exhibited strong antibiofilm activity after 48 h of exposure. The adhesion strategy employed here is PAMAM coating, and the binding capacity was assessed in vitro, and after several rinses with deionized water, PAMH was still present in enamel samples, indicating that the formulation was not washed off. In vitro models of remineralization suggested a potent remineralization activity after 3 days of treatment, where PAMH was capable to promote the reconstruction of exposed dentine. In vivo rat model was used to investigate the anti-cariogenic properties of PAMH, and results indicate in vivo biocompatibility and a significant decrease in pro-cariogenic parameters, suggesting a good in vivo anti-cariogenic effect. Together, these results represent a great opportunity for novel materials for enamel repair.

Zhu and co-workers also explored the potential of plant-derived molecules in dentistry [56]. The authors developed a PAMAM-modified phosphorylated dendrimer loaded with apigenin, a natural flavonoid, for simultaneous remineralization and antibacterial purposes. After 4 weeks of treatment using the dendrimer, in vitro dentine slices showed almost full occlusion of dentine tubules. The formulation also showed anti-biofilm activity (after 48 h exposure) against *S. mutans* and other microbes present in biofilms. In vitro cytocompatibility was confirmed using L929 cells, and results indicate a biocompatible non-toxic formulation. In vitro, adhesive properties were investigated using a human dentine sample and demonstrated strong bonding between dendrimer and dentin. The dendrimer showed sustained release of apigenin for up to 96 h. This work represents an advance toward the understanding of new materials capable to stop disease progression and impair future degradation by bacterial infections, as promising candidates for clinical therapy.

The anti-biofilm properties of dendrimers were investigated by Backlund and coworkers [57]. The authors developed and characterized alkyl chain-modified nitric oxide-releasing dendrimers. The in vitro anti-biofilm properties were investigated using planktonic and *S. mutans* biofilm (ATCC 27517) models. The polymer PAMAM was used to generate several alkyl-modified dendrimers. The most hydrophobic dendrimers increased bactericidal and anti-biofilm activity, as a result of enhanced dendrimer–bacteria interaction (due to high hydrophobicity), confirmed by confocal microscopy. Among the tested polymers, octyl- and dodecyl-modified PAMAM showed the strongest anti-biofilm activity. In vitro cytotoxicity was investigated using mammalian cells—human gingival fibroblasts (HGF-1), and the formulation presented significant toxicity to these cells. However, the addition of nitric oxide to octyl- and dodecyl-modified dendrimers decreased their toxicity. Together, these results reinforce the antimicrobial and anti-biofilm properties of dendrimers in dentistry, as a tool to eradicate dental infections and their resistance forms, as biofilms.

#### 3.1.7. Micelles

Polymeric micelles (PMs) are kinetically stable self-assembled monolayered core–shell vesicles (10–100 nm) made of polymers (i.e., surfactants) that can encapsulate both hydrophobic and hydrophilic drugs. The general structure of a micelle is composed of a hydrophobic core and a hydrophilic surface. The most commonly used polymers are poloxamers (amphiphilic block copolymers) and polyethylene glycol (PEG). Some advantages of micelles are the ability to improve the solubility and stability of poorly water-soluble drugs, promote a sustained drug release, improve permeability, and biocompatibility, in addition to improved drug biodistribution, that can be achieved by surface modifications (i.e., acrylate groups, which increases the mucoadhesion), as well as reduced side effects [108,109]. Common uses of PMs in dentistry include their use for the protection of dental structures, in combination with antimicrobial drugs for the inhibition of demineralization (Table 3) [110].

Yi and coworkers developed adhesive PMs containing farnesal (Far) for the prevention and treatment of dental caries [83]. Far is a natural product present in propolis and citrus fruit, with potent bactericidal activity but low water solubility, which is a limiting factor to the treatment of buccal diseases. Far was conjugated with polyethylene glycol (PEG), and with pyrophosphate (PPi), responsible for the adherence to the tooth enamel, and subsequentially loaded into mPEG-PLA PMs. The PMs presented narrow size distribution (~146 nm) with a polydispersity index (PDI) of 0.234, as well as negative zeta potential (ZP). Far release in acidic pH (4.5) was more than 2.0 times faster than in pH 7.4, desirable to achieve maximum drug concentration on bacteria-infected tissues. The formulation containing PPi properly binds to hydroxyapatite in vitro within 2 min of exposure, which authors compare as equivalent to brushing the teeth or using mouthwashes. In vivo, using a Sprague–Dawley rat model, the anti-cariogenic effect of PMs was found to be higher than free Far, as confirmed by superior molar mechanical strength, and anti-demineralization effect. These results are important to a better comprehension of drug delivery systems capable of properly adhering to hydroxyapatite and promoting fast release under the acidic microenvironment of dental plaque, and repurposing the clinical use of poorly water-soluble substances in caries treatment.

Tooth-binding PMs were also studied by Chen and coworkers [84] aiming to enhance the anti-biofilm activity of the anti-bacterial drug triclosan. The PMs were composed of modified Pluronic copolymers and alendronate (ALN). ALN is a mineral binding moiety that was chemically conjugated to Pluronic. The PMs with triclosan showed a narrow size (~53 nm) and polydispersity index of 0.270. PMs containing Pluronic 123 showed in vitro hydroxyapatite fast binding (>20% at 10 min of exposure). The formulation presented sustained in vitro release for up to 48 h. The PMs containing ALN and Pluronic 123 showed the strongest in vitro anti-biofilm action against *S. mutans* (UA159), compared to untreated control, and the highest bacteria-killing effect on preformed *S. mutans* biofilms. This work makes an important reflection on the need for bioadhesive properties for a more assertive and effective treatment of bacterial infections in dentine, and how this is directly related to the development of the drug delivery system, being necessary to employ ingredients that interact quickly and strongly with hydroxyapatite. Surface modifications made to nanocarriers can also increase interaction with the target pathogen by increasing hydrophobic interactions, as in the case of ALN.

Xu and coworkers [85] successfully developed a dental plaque-inspired micellar nanosystem to treat caries and to act as a restorative biomaterial, encapsulating tannic acid (see anti-bacterial properties [111,112]) and sodium fluoride (NaF). After obtention, PMs were chemically conjugated to a salivary-acquired peptide (SAP), as a strategy to increase enamel adhesion. PMs have a small size (300 nm) with homogenous size distribution (0.12). The PMs presented in vitro pH-responsive release, in acidic media. The nanosystem was able to efficiently adhere to human enamel samples in vitro for over 2 days of exposure to artificial saliva, which is mandatory for clinical applications. The formulation presented strongly in vitro inhibition of *S. mutans* (UA159) in acidic pH, and diffusion ability into cariogenic acidic biofilms. The dual drug-loaded PMs showed a superior remineralization effect compared to free drugs, highlighting its potential as a restorative biomaterial and suggesting the importance of proper adhesion to enamel for promoting remineralization. The formulation showed good biocompatibility to healthy cells (ATCC MC 3T3, osteoblast precursor cell line) and no impact on oral microflora quali-quantitative composition, reinforcing its clinical potential. Using an in vivo Sprague–Dawley cariogenic model, the authors investigated the efficacy of PMs against dental caries and as a restorative biomaterial. Results indicate a significantly higher anti-cariogenic effect with reduced number and severity of bacterial infections, compared to conventional treatment using chlorhexidine. The PMs also showed anti-demineralization effect, preventing in vivo tooth decay. This work is an important reference of combined strategies to treat both caries infections and tissue damage, preventing further demineralization.

PMs can also be used combined with another therapeutic approach, as in the case of antimicrobial photodynamic therapy (aPDT) for the treatment of oral biofilms. The aPDT is still a challenge because many photosensitizers are big and insoluble molecules, that cannot adhere to human dentine, compromising the treatment’s effectiveness. PMs can be particularly useful in aPDT since they can increase the solubility and dispersion of insoluble drugs such as curcumin, and also increase the retention time in the oral cavity, because they improve the interaction with dentine and with bacteria, such as *S. mutans.* Dantas Lopes dos Santos designed and investigated the biological properties of Pluronic F127-based PMs containing curcumin as a photosensitizing drug [113]. The PMs showed a small size (<100 nm) and a stability in an aqueous medium for up to 30 days. Using in vitro *S. mutans* (ATCC 25175) and *Candida albicans* (ATCC 18804), the authors proved the ability of PMs in the presence of light to reduce the viability of both microorganisms’ biofilms. Together with other data discussed in this section, such as those regarding biocompatibility and good adhesive properties, PMs represent a promising approach for the treatment of dental caries in humans, and the effect can be enhanced in resistant bacterial infections (such as biofilms) using aPDT.

### 3.2. Inorganic Nanoparticles

#### 3.2.1. Silver Nanoparticles

Silver nanoparticles (AgNPs) have been shown to be highly active against microorganisms in general. Its antibacterial action is highly explored in the medical field, standing out in the dental field due to its properties in reducing the formation of biofilms as well as in the treatment of caries (Table 4). The synthesis of AgNPs can be occur from different processes: chemical, physical, and biological synthesis. The physical process is the most expensive and the NPs presented agglomeration [114,115,116]. The chemical and biological are the most common, in which the AgNPs obtained exhibited a large range of particle sizes and shapes. The chemical process is less eco-friendly than the biological; however, the chemical process is more reproducible [117]. The biological obtainment stands out in terms of the stability of the particles formed [114,115,116]. AgNPs can be surface-modified to increase their biocompatibility, intracellular uptake for drug delivery, and longer stability. Copolymers has been used in AgNPS synthesis with the purpose of reducing and stabilizing agent.

The broad antibacterial activity of AgNPs against both Gram-positive and Gram-negative bacteria is the reason that these nanoparticles are so advantageous in the dental area. The action of AgNPs is related to the interference of cell wall synthesis and also interfering in the replication and transcription process [29,114,115].

Xiaoxue Yin and coworkers (2020) performed an interesting review that highlighted, in the literature, that 89% of studies involving silver nanoparticles investigated their antibacterial properties, and they all found evidence about the Ag nanomaterials in the inhibition and growth of cariogenic bacteria. The main mentioned bacteria are *S. mutans* [129]. This same author investigated the remineralizing and staining effects of sodium fluoride (NaF) with AgNPs on artificial dentine caries. The authors performed an investigation in human dentine blocks with artificial caries. They were divided into four groups in which received NaF solution, NAF in AgNPs, only AgNPs, and negative control. The AgNPs was prepared by chemical process, but the authors used epigallocatechin gallate as a reducing agent and chitosan as a dispersant. The results indicated that NaF solution in AgNPs can remineralize without staining, and also, only AgNPs exhibited activity [118].

Al-Ansari and coworkers (2021) synthetized AgNPs with Arabic gum and evaluated their activity on *S. mutans.* The AgNPs inhibited the genes responsible for biofilm formation of *S. mutans* over host teeth and gums and also inhibited virulent protective factors and survival promoter genes [130]. Jasso-Ruiz coworkers (2020) studied in vitro bacterial inhibitions by AgNPs. The study was based in 300 orthodontic brackets (150 brackets for *S. mutans*, 150 brackets for *S. sobrinus*) classified into groups with silver nanoparticles and groups without silver nanoparticles. The results showed the importance of silver coating to decrease the adhesion of both *S. mutans* and *S. Sobrinus* to the orthodontic brackets. Further, the modification of the surface of orthodontic brackets with silver nanoparticles can modify the development of dental plaque and consequently, dental caries, which demonstrates their antibacterial properties [131].

Espinosa-Cristóbal and coworkers (2019) evaluated the antimicrobial AgNPs activity in oral microbiomes clinically isolated from young and young-adult patients. The AgNPs used in the antibacterial assay for MIC determination presented about 10–30 nm in size, −50 mV surface charge, and spherical shape. The results showed no statistical difference in the MIC value between female and male group. However, some female biofilms showed resistance in the presence of 30 nm AgNPs compared to smaller NPs. The results suggest that the Ag concentrations for both sizes of AgNPs maintained a uniform antimicrobial activity at any age or gender. However, male patients showed MIC values that could increase according to the age [119].

Jiménez-Ramírez and coworkers (2021) used in their study AgNPs based in the previous mentioned study of Espinosa-Cristóbal and coworkers (2019) [132,133]. The AgNPs were prepared by chemical process and presented about 10 nm and 38 nm, with −10 to −30 mV surface charge, and spherical and pseudospherical shape. In Jiménez-Ramírez and coworkers’ (2021) studies, the MIC was determined by the action of AgNPs in biofilms from patients with and without dental caries (30 subjects each). PCR was performed to determine the presence of oral bacteria related to dental caries and determined *S. mutans* and *S. sobrinus* strains, principally. AgNPs had significant antimicrobial effects against all samples of dental plaque [115].

AgNPs are nanocomposites with extensive dental application, and can be applied not only when there is a caries process installed but can also be used in the production of prostheses, implants, seeking to avoid the formation of biofilms. Furthermore, AgNPs were shown to be efficient in helping to promote remineralization without staining. Thus, this is a highly promising system in the dental field and stands out from other nanosystems used [29].

#### 3.2.2. Zinc Nanoparticles

Zinc oxide nanoparticles (ZnO NPs) have unique properties, such as several morphologies, large surface area to volume ratio, potent antibacterial activity, and biocompatibility. This range of features makes this system attractive and easy to be managed for use in controlling biofilms. Zinc nanoparticles have applications reported in the literature in several areas, such as central venous and urinary catheters, prosthetic joints, cardiovascular implantable devices, contact lenses, intrauterine contraceptive devices, breast implants, and dental implants (Table 4) [134].

To effectively treat a biofilm, the nanoparticle must be able to access the biofilm interface and attach itself to the outer surface of the biofilm. Then diffuse through the matrix which can be affected by nanoparticles features, such as size, surface charge, shape, and hydrophobicity [31,135]. ZnO NPs are considered versatile nanosystems and they presented an attached feature suitable to develop the diffusion by the biofilm matrix and act as an antibacterial generating ROS and disrupting the membrane traffic [30,31].

Several physicals, chemical, and biological methods used to produce uniform ZnO NPs were reported, such as solvent-based ultrasonic irradiation, hydrothermal, microemulsion, physical vapor deposition, thermal evaporation, solvothermal, and microwave synthesis [136]. The method of preparation of ZnO NPs will determine their antimicrobial activity. In this way, different shapes and types of ZnO structures, such as nanorods, nanopowders, nanotubes, nano/microspheres, and quantum dots, can exhibit certain antimicrobial activity [137].

Biochemically, the antibacterial property of ZnO NPs is close to AgNPs due to their reduced sizes, large surface area, and accumulation behavior. However, it has already been mentioned in the literature that the antibacterial capacity of ZnO NPs may be greater than that of Ag NPs due to their capacity to react with more molecules per unit of surface. However, in vitro, these differences are not always observed [30].

Mirhosseini and coworkers (2019) studied antimicrobial effect of various sizes and concentrations of ZNO NPs on several microorganisms including oral strains. The activity was observed by disk diffusion method. The greatest inhibition was observed for *S. mutans* with particles about 20–40 nm. The increase of size demonstrated a decrease of inhibition of *S. mutans* [138]. Several authors found a great antimicrobial activity of ZnO NPs in this same size range against oral strains [137]. Recently, the ZnO NPs and related nanoparticles were investigated against *S. mutans.* Among the studied NPs, the authors found a high activity for ZnO NPs; however, the Ca_19_Zn_2_(PO_4_)_14_ NPs exhibited half of the MIC value found for ZnO NPs. Thus, the authors presented the possibility of coating dental surfaces [120].

Furthermore, the incorporation of ZnO NPs into toothpastes to treat gingivitis has been carried out since their anti-inflammatory activities were elucidated. ZnO NPs can block the production of pro-inflammatory cytokines such as IL-1 and IL-18, and also inhibit mast cell proliferation and suppress lipopolysaccharide-induced cyclooxygenase-2 and inducible nitric oxide synthase expression [30].

#### 3.2.3. Calcium Nanoparticles

Calcium nanoparticles are inorganic nanoparticles whose matrix is formed by calcium [34,139,140,141]. Calcium nanoparticles confer biominerals mechanical strength and self-preservation in biological fluids by preventing ion depletion during bacteria-induced tooth demineralization. It exhibits biocompatibility and low toxicity due to the chemical similarity with these tissues. They have a predictable metabolism, reducing problems of metabolic toxicity. They are resistant to microbiological degradation when compared to lipid and polymeric systems. Calcium particles, mainly phosphate, constitute the bones and the teeth. Despite this, ion delivery to suppress demineralization has been a challenge for decades due to the difficulty of local delivery. In this scenario, calcium nanoparticles are able to overcome this drawback [33,125,142,143].

The obtention of calcium nanoparticles with irregular size and shape is the main problem. The controlled synthesis of these nanoparticles is an important parameter for dental application due to biomimetic use, since biomineralization crystals have different sizes and shapes according to the body tissue [142,144]. The use of polyelectrolytes or charged adsorbent molecules are strategies used to limit the space during the nucleation process in aqueous solution, controlling the size of the nanoparticles and forming stable colloids [144,145].

Calcium-based ion delivery nanosystems have been reported as promising agents for saturation of these ions in the oral environment, preventing dental caries and increasing remineralization, such as calcium carbonate (CaCO_3_) (Table 4) [139], calcium phosphate (Ca(H_2_PO_4_)_2_) [146], tricalcium phosphate (Ca_3_(PO_4_)_2_) [124], hydroxyapatite (Ca_5_(PO_4_)_3_OH), and amorphous calcium phosphate nanoparticles [125,147]. Calcium nanoparticles have a higher amount of calcium when compared to oral health products with added calcium ions. However, the efficiency in the process of remineralization and dentin occlusion is associated with the intrinsic characteristics of these nanoparticles and the mode of use of the products containing the nanoparticles [142,144,148].

The antibacterial activity of a nanocomplex against *S. mutans* growth and its remineralizing potentials on demineralized bovine enamel was evaluated by Elgamily and coworkers (2019). They demonstrated that the associated use of different nanoparticles, including calcium phosphate nanoparticles, enhances the anti-cariogenic and remineralizing effects. The nanocomplexes tested were composed of nano amorphous calcium phosphate (nACP), nano casein phosphopeptides (nCPP), probiotic—*Lactobacillus rhamnosus* B-445, and nano glycomacropeptide (nGMP). The treatment with the paste containing the three associations (nCPP + nACP + *L. rhamnosus*, and nCPP + nACP + nGMP) showed a higher mean zone of inhibition than the nCPP and nACP group for *S. mutans.* It also showed significant mean values of remineralization in the 15-day period, as well as deposition of nanometer particles on the enamel surface pattern demonstrated by SEM analysis [122]. The results show the long-term protective effect of nanocomplex, probably due to the accumulation of ions on the teeth, which causes a buffer state owing to supersaturation. However, casein phosphopeptides and amorphous calcium phosphate have a slightly lower potential in the remineralization of early enamel caries compared to fluoride [149,150]. Nevertheless, these nanoparticles can be incorporated into dental products as a caries-preventive agent, acting in synergy with fluoride and also carrying agents with antimicrobial effects, such as antimicrobial toothpaste containing casein phosphopeptides nanoparticles loaded with a probiotic strain [122].

Hydroxyapatite nanoparticles are integrated into oral care products to promote enamel remineralization by ion supersaturation at the lesion, a mechanism similar to other calcium nanoparticles. However, these nanoparticles promote enamel regeneration by forming biomimetic film similar to biological hydroxyapatite, proving to be more effective in caries repair in vitro when compared to fluoride and casein nanoparticles. This occurs because the remineralization layer is resistant to brushing due to the chemical bonds between the synthetic and natural crystals of the glaze, even at a potentially cariogenic pH (pH 4) [143,151,152].

A mouthwash based on hydroxyapatite nanoparticles (130 nm) was evaluated for its anti-adhesive and antibacterial effect on bacterial biofilm. The activity was evaluated in situ using a specimen fixed to the mandible of nine volunteers for 8 h after using the hydroxyapatite mouthwash, water only, or a 0.2% chlorhexidine solution. Through fluorescence staining using DAPI, Concanavalin A, and BacLight LIVE/DEAD it can be seen that both types of mouthwashes showed a significant reduction in vital bacteria on the surface of the specimens compared to water, but no significant difference between them. Similarly, the detection of glucan was reduced, demonstrating the decrease in the extracellular matrix and biofilm-forming ability. Despite not showing a reduction in the adhesion capacity, the hydroxyapatite-based mouthwash was similarly effective to chlorhexidine in reducing biofilm-forming bacteria. It occurs probably due to the accumulation of crystalline hydroxyapatite micro agglomerates (100–500 nm) around the bacteria, which happens possibly by the interactions between the hydroxyapatite particles and bacterial fimbriae, as demonstrated by transmission electron microscopy analysis of saliva samples obtained directly after rinsing [123].

These nanoparticles can be complexed with ions associated with the remineralization and anti-erosion process (e.g., fluoride, stannous, and phosphate) for synergistic effects on calcium deposition. Fluoride and stannous functionalized β-tricalcium phosphate nanoparticles (β-TCP) showed to be effective in controlling dentin erosion in vitro when specimens were immersed in treatment solution two times daily for 5 days. The higher calcium and fluoride availability of the β-TCP nanoparticles resulted in approximately two-fold less specimen surface loss compared to the control (water) and one-fold less compared to fluoride solution, stannous solution, and solution of fluoride- and stannous-functionalized β-TCP nanoparticles. Furthermore, β-TCP functionalized with fluoride was able to increase the deposition of the ion on the eroded dentin substrate and showed greater protection of the dentin against the acidic environment, which was favored by the small size (150–300 nm), spherical shape, and higher amount of calcium and fluoride ions, confirmed through transmission electronic microscopy, energy dispersive spectroscopy, and X-ray diffraction [124].

Amorphous calcium phosphate nanoparticles complexed with fluoride ions were evaluated for their anti-carious and remineralizing properties to overcome the instability of calcium phosphate in aqueous media by delaying crystalline phase formation, improving their performance, and facilitating their use. The spherical nanoparticles with sizes from 20 to 50 nm were able to maintain their composition and amorphous nature after complexation with fluorine for up to one year according to transmission electronic microscopy, energy dispersive spectroscopy, X-ray diffraction, and Fourier transform infrared results. The ability to gradually release the ions with subsequent local supersaturation and remineralization was evaluated in vitro in acidified artificial saliva. The functionalized nanoparticles showed time-dependent conversion to crystalline. They were efficient in gradually releasing calcium and fluoride ions, 5 to 20% in 2 h, depending on the citrate concentration used in the synthesis process. In vitro remineralization and occlusion of dentinal tubules showed the ability of nanoparticles to adhere to tooth surfaces. Initially, injured human enamel and dentin samples were treated for 24 h with concentrated pastes containing nanoparticles or nanoparticles complexed with fluoride. Calcium nanoparticles had a higher degree of tubule occlusion compared to functionalized nanoparticles due to a lower conversion rate to hydroxyapatite. Functionalized nanoparticles produced with lower citrate concentration showed the lower occlusive capacity of dentin and a higher amount of crystalline microparticles. In the enamel, remineralization is observed by scanning electron microscopy nano or microcrystals deposited in a less orderly way than the original crystals, but that can guide a biological remineralization process making it more efficient [125].

#### 3.2.4. Titanium Nanoparticles

Titanium dioxide (TiO_2_) has high physical and chemical stability and excellent biocompatibility. It exhibits antibacterial activity against several strains, including *S. mutans*, the cause of dental caries (Table 4) [153,154]. The antibacterial activity may be associated with its photocatalytic ability, whereby it releases reactive oxygen species causing lipid peroxidation of cell membranes, inhibiting bacterial cell functions, and leading to bacterial death. In addition, TiO_2_ can improve the mechanical properties of other materials, such as increasing the compressive strength of glass ionomer cement (GIC) [37,153,155]. Titanium dioxide nanomaterials have been widely used to exploit the intrinsic properties of titanium dioxide and nanoscale materials. Titanium dioxide nanostructures include titanium dioxide nanoparticles and titanium dioxide nanotubes. Nanoscale titanium dioxide has demonstrated application potential in several areas, including the surface modification of dental implants and additives of dental materials [156].

TiO_2_ nanoparticles synthesized by Bacillus subtilis were used to produce a new GIC restorative material for dental caries treatment. The nanoparticles showed to be biocompatible and non-cytotoxic. The lowest cell viability (91.71%) was observed on day 21 with no change in fibroblast morphology. There was an increase in compressive strength and a decrease in flexural strength of GIC with up to 5% inclusion of TiO_2_ nanoparticles, changing negatively and progressively with increasing nanoparticle concentration. GIC containing 5% titanium nanoparticles exhibited lower porosity and micro-cracks, which may be associated with the better mechanical properties of the GIC. Bacillus subtilis-derived TiO_2_ nanoparticles show potential for developing new restorative materials for dental use [126]. Other studies demonstrate the potential of these nanoparticles to increase the mechanical strength of polyvalent restorative material since GICs are brittle due to their low cohesive strength [127]. Araújo and coworkers demonstrated that 5% TiO_2_ nanotubes incorporated into GIC presented higher antibacterial properties against *S. mutans* compared to 3% and 7% TiO_2_ nanotubes, regardless of the in vitro treatment time. GIC with 5% nanoparticles affected the morphology and organization of *S. mutans*, probably due to reduced expression of covR when compared with GIC with nanoparticles, enhancing the anti-cariogenic properties of GIC [128]. Another study also reported superior results for antimicrobial activities on *S. mutans* and mechanical strength of GIC containing 3% (w/w) titanium nanoparticles [35]. Similar results were observed by Sodagar and coworkers, in which an increased inhibition of Streptococcus strains were observed with the incorporation of 10% titanium nanoparticles in the resin material, Transbond XT composite, different from GIC [36]. Titanium nanoparticles are shown to be nanomaterials capable of providing antibacterial properties without reducing mechanical properties and are promising for future applications in dental restorative materials.

## 4. Conclusions

Despite the advantages in the medical field, the prevention and treatment of dental caries remain being a challenge, given that oral biofilms have a complex architecture that makes difficult the permeation of several treatments. Furthermore, enamel remineralization is a slow process, which is an essential step for a complete dental treatment. In this review, we focused on studies that investigated drug delivery systems to prevent the initial formation of oral biofilms, especially the adhesion of *S. mutans*, to remove already formed biofilm, and to accelerate the remineralization step.

In a general way, the use of mucoadhesive systems, such as mucoadhesive liquid crystalline systems, are an important strategy for increasing the permanence time of these systems in the teeth. This property allows the drug to act for a longer period to protect the teeth against the adhesion of microorganisms and make possible complete enamel remineralization. Furthermore, the use of mucoadhesive formulations and in situ gels, prolonged the time of these systems on teeth and they can improve drug permeation into the biofilm, which is one of the main difficulties of the currently available treatment and responsible for biofilm resistance. In addition, several drug delivery nanosystems have demonstrated a great ability to permeate biofilms due to their low particle size, besides being able to be produced with a positive target to improve their interaction with the negative surface of bacteria and with a different shape to enhance their permeation. Finally, various types of drug delivery systems have shown to be promising in the prevention and treatment of caries, especially those that have the ability to inhibit biofilm formation and growth and promote the remineralization process, such as fluoride-loaded calcium phosphate nanoparticles, dendrimers, and liposomes. However, the majority of research regarding the potential application of these drug delivery systems in dentistry using in vitro models and few clinical trials are available, mainly with the application of lipid- and polymer-based systems. Thus, it is expected that in the near future, more clinical studies will be carried out to understand better the effect of each system on preventing and treating oral caries.

## Figures and Tables

**Table 1:** Advantages and limitations of different types of drug delivery systems or dental application.

Drugs Delivery System	Advantages	Limitations	Ref.
Liquid crystalline system	Low cost Easily scalable Controlled release Increased retention time in the mouth Stimuli-responsive properties (e.g., in situ gelling properties)	Time required for LCS in contact with buccal mucosa to develop bioadhesive properties and release the active	[16,17,18]
Liposomes	The morphology and composition can influence the erosion and demineralization process Charged liposomes can influence the remineralization process by forming complexes with minerals pH-sensitive liposomes can increase drug release in the mouth	Limited storage conditions Short retention of anionic and neutral liposomes	[19,20]
Nanoemulsion	Thermodynamic stability High tissue penetration ability	High surfactant amount, its choice influences the toxicity	[21,22]
Polymeric nanoparticles	Biocompatible Biodegradable Avoids leakage of the drug Mucoadhesion depending on the polymer used (e.g., chitosan)	Difficult to scale up	[23,24]
Hydrogel	Biocompatible of some polymers Mucoadhesion depending on the polymer used (e.g., chitosan)	Lack of biocompatibility of some polymers Low tensile strength Difficult in loading hydrophobic drugs	[25,26]
Dendrimer	Intrinsic bioadhesive properties Easily tunable Easily scalable Controlled release	Limited entrapment efficacity compared to other colloidal carriers Possible intrinsic toxicity	[27]
Polymeric micelles	Easy obtention Easily surface modification	Possible intrinsic toxicity	[27]
Silver nanoparticles	Broad antibacterial activity against Gram-positive and Gram-negative bacteria Low bacterial resistance High penetration biofilm penetration	Change in color of dental materials	[28,29]
Zinc nanoparticles	Antibacterial activity by generating reactive oxygen species (ROS) and disrupting the membrane traffic	Potential toxic effect depending on concentration/dose, time of exposure, and size	[30,31,32]
Calcium phosphate nanoparticles	They promote self-preservation of biominerals when in contact with saliva. They are biomimetic and therefore biocompatible, with low toxicity and predictable metabolism. They increase the saturation of biominerals at the site of action.	The obtention of nanoparticles with varying sizes and shapes is the main problem because it impairs the biomimetic function. However, a controlled nucleation process solves the problem.	[33,34]
Titanium nanoparticles	They enhance the antimicrobial properties of titanium dioxide due to the increased contact surface area associated with the smaller size. They improve the mechanical properties of materials used for temporary cavity sealing, such as increasing the compressive strength of glass ionomer cement (GIC).	The mechanical strength properties change negatively or progressively according to the concentration of nanoparticles added to the materials.	[35,36,37]

**Table 2:** Summarization of clinical trials for dental caries adopting nanoparticles. Data obtained from ClinicalTrials.gov.

Study Title	Identifier	Nanoparticle	Aim	Last Update
Clinical Evaluation of Silver Nanoparticles in Comparison to Silver Diamine Fluoride in Management of Deep Carious Lesions	NCT05231330	Silver	To evaluate the effect of fluoride varnish with silver nanoparticles in comparison to silver diamine fluoride.	9 February 2022
Effect of the incorporation of copper and zinc nanoparticles into dental adhesives	NCT03635138	Copper and Zinc	To study if the addition of copper or zinc nanoparticles to a dental adhesive confers antimicrobial and enzymatic degradation-resistant properties, retaining its adhesion mechanical properties and biocompatibility.	17 August 2018
Evaluation of the antibacterial effect of laser diode and zinc oxide nano particles in cavity disinfection	NCT03478150	Zinc Oxide	To evaluate the antibacterial effect of laser diode and zinc oxide nano-particles when used as cavity disinfectants	27 March 2018
Nanosilver fluoride to prevent dental biofilms growth	NCT01950546	Silver	To evaluate the effectiveness of nanosilver fluoride for controlling the growth of S. mutans present in dental plaque of children.	10 June 2015
Antibacterial effect of nano silver fluoride vs chlorhexidine on occlusal carious molars treated with partial caries removal technique	NCT03186261	Silver	To evaluate the effect of silver nanoparticles in comparison with Chlorhexidine on Occlusal Carious Molars regarding the removal of bacterial plaques.	16 September 2021
Antibacterial effect and clinical performance of chitosan modified glass ionomer	NCT04365270	Polymeric and Titanium dioxide	To assess the clinical success and the antibacterial effect on carious dentine of glass ionomer when modified with Chitosan and/or Titanium dioxide nano particles vs the control group of modification with Chlorhexidine as control when used in primary molars.	12 January 2021
Clinical performance and wear resistance of two nano ceramic resin composite in class I cavities	NCT04738604	Ceramic resin	Tooth restorations.	31 August 2021
Remineralization of early carious lesion using natural agents versus bioadhesive polymers	NCT04390256	Bioadhesive Polymers	Remineralization.	15 May 2020
Cariostatic and remineralizing effects of three different dental varnishes	NCT04887389	Silver nanoparticles in varnishes	To evaluate the cariostatic and re-mineralizing effects of Nano silver fluoride, Nano Hydroxyapatite and sodium fluoride varnishes in caries prevention.	8 June 2022
P11-4 and nanosilver fluoride varnish in treatment of white spot carious lesions	NCT04929509	Silver	To evaluate the biomimetic remineralization of initial carious lesions as a minimal invasive therapy using Self-Assembling Peptide P11-4 (Curodont Repair) which enhances remineralization of white spot lesions.	18 June 2021

**Table 3:** Recent studies that used organic drug delivery systems for caries prevention and treatment.

Nanosystem	Drug	Composition	Study Model	Effect	Ref.
Liquid crystalline system	β-defensin-3 peptide fragment	Carbopol^®^ 974P, Carbopol^®^ 971P, polyoxypropylene-(5)- polyoxyethylene-(20)-cetyl alcohol, and oleic acid	In vitro	The developed formulation showed a cumulative effect against *S. mutans*.	[15]
Liquid crystalline system	p1025 peptide	Polyoxypropylene-(5)-polyoxyethylene-(20)-cetyl alcohol and tea tree oil	In vitro	The liquid crystalline systems showed shear thinning and thixotropy characteristics favorable for treatment of dental caries.	[8]
Liquid crystalline system	p1025 peptide	Polyoxypropylene-(5)-polyoxyethylene-(20)-cetyl alcohol, oleic acid, and Carbopol^®^ 974P	In vitro	Reduced *S. mutans* biofilm formation with a limited cytotoxicity in human epithelial cells (HaCaT).	[64]
Liquid crystalline system	Curcumin	Polyoxypropylene-(5)-polyoxyethylene-(20)-cetyl alcohol, oleic acid, and Carbopol^®^ 974P	In vitro	Reduced significantly the log_10_ when photodynamic therapy was applied.	[65]
Liposomes	Nisin	Dipalmitoylphosphatidylcholine and phytosphingosine	In vitro	Cationic nisin-loaded liposomes showed greater antimicrobial activity against *S. mutans* than neutral and anionic liposomes.	[66]
Liposomes	Doxycycline	Lecithin	In vitro	Doxycycline-loaded liposomes removed the biofilm from the hydroxyapatite surface.	[67]
Liposomes	Demineralized dentin matrix	Phosphatidylcholine, Phosphatidylserine, and cholesterol	In vitro	Activated the dental tissue repair in vitro.	[68]
Nanoemulsion	Cetylpyridinium chloride	Soybean oil and Triton X-100	In vitro	Nanoemulsion showed greater inhibitory effect against microorganisms than chlorhexidine.	[69]
Nanoemulsion	Chlorhexidine acetate	Tween 80, propylene glycol, and isopropyl myristate	In vitro and in vivo	Nanoemulsion significantly reduced oral biofilm and inhibited biofilms formation in rats.	[70]
Nanoemulsion	Cinnamon essential oil	Cocamidopropyl betaine and cinnamon essential oil	In vitro	The developed formulation inhibited the maturation and growth of oral biofilms.	[71]
Polymeric Nanoparticle	-	Chitosan and tripolyphosphate	In vitro	Chitosan nanoparticles decreased the cell viability of *S. mutans* and *C. albicans*.	[72]
Polymeric Nanoparticle	-	Chitosan	In vitro	Decreased biofilm formation of *S. mutans* by 88.4%, *S. salivarius* by 93.4%, *S. sobrinus* by 78.9%, and *S. sanguis* by 72.6%.	[73]
Polymeric Nanoparticle	Chloroaluminum phthalocyanine	Chitosan	In vitro	The photodynamic mediated by the developed system significantly reduced *S. mutans* UFC	[74]
Hydrogel	QPs peptide	Chitosan	In vitro	Decreased biofilm formation of *S. mutans* by approximately 100%.	[75]
Hydrogel	Mentha piperita	Chitosan	In vitro	Decreased biofilm formation of *S. mutans* by 57%.	[76]
Hydrogel	Tormentil	Carboxymethylcellulose	In vitro	Total *S. mutans* biofilm inhibition using 2 mg/mL of the tormentil.	[77]
Hydrogel	Methylene blue	Papain	In vitro	Methylene blue-loaded papain hydrogel showed a reduction in 2-log CFU	[78]
Dendrimer	Triclosan	Carboxyl-terminated PAMAM polymer	In vitro	In vitro remineralization of human dentine, adhesive properties, and sustained release.	[79]
Dendrimer	Honokiol	Carboxyl-terminated PAMAM polymer	In vitro and in vivo	In vitro sustained release and remineralization, adhesive properties, anti-biofilm action, and in vivo anti-cariogenic activity	[80]
Dendrimer	Apigenin	Phosphate ester-terminated PAMAM dendrimer	In vitro	In vitro sustained release, induced remineralization, antibiofilm activity, adhesive properties, biocompatible	[81]
Dendrimer	Nitric oxide	Octyl- and dodecyl-modified PAMAM	In vitro	Increased in vitro antibiofilm action and fast release, at acidic pH. More hydrophobic formulations showed increased dendrimer-bacteria interaction	[82]
Polymeric micelles	Farnesal	mPEG_2000_-PLA_2000_	In vitro	Fast adhesion to hydroxyapatite and pH-triggered release in acidic pH, in vitro anti-demineralization, and in vivo anti-cariogenic properties	[83]
Polymeric micelles	Triclosan	ALN-modified Pluronic copolymers	In vitro	Fast and strong binding to hydroxyapatite in vitro, anti-biofilm, and strong killing effect against *S*. *mutans*, sustained release	[84]
Polymeric micelles	Tannic acid and NaF	3-maleimidopropionic acid-poly(ethylene glycol)-block-poly(l-lysine)/phenylboronic acid (MAL-PEG-*b*-PLL/PBA) and SAP	In vitro and in vivo	pH triggered release, in vitro biocompatibility, strong enamel adhesion, anti-biofilm activity, and anti-demineralization activity. In vivo anti-cariogenic effect superior to conventional treatment and remineralization properties	[85]

**Table 4:** Recent studies that used inorganic drug delivery systems for caries prevention and treatment.

Nanosystem	Composition	Study Model	Effect	Ref.
Silver nanoparticles	Silver nitrate and gallic acid	In vitro	Silver nanoparticles exhibited great antimicrobial activity against dental plaque.	[115]
Silver nanoparticles	Silver nitrate, epigallocatechin gallate, and chitosan	In vitro	The developed formulation can remineralize dentine caries	[118]
Silver nanoparticles	Silver nitrate and gallic acid	In vitro	Silver nanoparticles showed antimicrobial activity against microorganisms from oral biofilms, including *S. mutans.*	[119]
Zinc nanoparticles	Zinc acetate dihydrate	In vitro	Zinc nanoparticles reduced biofilm formation.	[120]
Zinc nanoparticles	Pure zinc block	In vitro	Rod-like shaped zinc nanoparticles exhibited greater inhibition on *Streptococcus sobrinus* and *S. mutans* compared with plate-like shaped nanoparticles.	[121]
Nanocomplex of calcium phosphate (nCPP), casein (nACP), probiotic and glycomacropeptide (nGMP)	nCPP, nACP, *L. rhamnosus* (10^9^ CFU/g) e nGMP, and toothpaste base.	In vitro	Increased remineralization, antibacterial effect, increased deposition on enamel surface with a long-term protective effect	[122]
Hydroxyapatite nanocrystals	Hydroxyapatite microclusters in bidestilled water	In vitro and in situ	Reduced the viable bacteria and glucans on the surface of specimens and increased interactions of hydroxyapatite particles and bacterial fimbriae.	[123]
β-tricalcium phosphate Nanoparticles	Calcium hydroxide, magnesium hydroxide, fluoride and/or stannous ions.	In vitro	Reduced enamel and dentin surface loss, improved anti-erosive effect	[124]
Fluoride-doped amorphous calcium phosphate nanoparticles	Calcium chloride dihydrate, sodium citrate tribasic dihydrate, sodium phosphate dibasic dihydrate, sodium carbonate monohydrate, sodium fluoride	In vitro	Decreased conversion to the crystalline phase in water, increased occlusion of dentinal tubules and enamel remineralization.	[125]
TiO_2_ Nanoparticles	TiO_2_ nanoparticles and glass-ionomer cements	In vitro	Increase in compressive strength and decreased in porosity and micro-cracks increasing mechanical strength.	[126]
TiO_2_ Nanoparticles	TiO_2_ nanoparticles and glass-ionomer cements	In vitro	Increased flexural strength, compressive strength, and diametrical tensile strength.	[127]
TiO_2_ nanotubes	TiO_2_ nanotubes and glass-ionomer cements	In vitro	Increased antibacterial property against *S. mutuans*, change in morphology and organization of *S. mutuans*, reduction in *covR* expression, increase in anti-cariogenic properties of glass-ionomer cements	[128]
